# *In Vivo* Analysis of Disease-Associated Point Mutations Unveils Profound Differences in mRNA Splicing of Peripherin-2 in Rod and Cone Photoreceptors

**DOI:** 10.1371/journal.pgen.1005811

**Published:** 2016-01-21

**Authors:** Elvir Becirovic, Sybille Böhm, Ong Nam Phuong Nguyen, Lisa Maria Riedmayr, Mirja Annika Koch, Elisabeth Schulze, Susanne Kohl, Oliver Borsch, Tiago Santos-Ferreira, Marius Ader, Stylianos Michalakis, Martin Biel

**Affiliations:** 1 Munich Center for Integrated Protein Science CIPSM, Ludwig-Maximilians-Universität München, München, Germany; 2 Department of Pharmacy–Center for Drug Research, Ludwig-Maximilians-Universität München, München, Germany; 3 Institute for Ophthalmic Research, Center for Ophthalmology, University of Tübingen, Tübingen, Germany; 4 Technische Universität Dresden, CRTD/DFG-Center for Regenerative Therapies Dresden, Cluster of Excellence, Dresden, Germany; National Eye Institute, UNITED STATES

## Abstract

Point mutations in peripherin-2 (PRPH2) are associated with severe retinal degenerative disorders affecting rod and/or cone photoreceptors. Various disease-causing mutations have been identified, but the exact contribution of a given mutation to the clinical phenotype remains unclear. Exonic point mutations are usually assumed to alter single amino acids, thereby influencing specific protein characteristics; however, they can also affect mRNA splicing. To examine the effects of distinct PRPH2 point mutations on mRNA splicing and protein expression *in vivo*, we designed PRPH2 minigenes containing the three coding exons and relevant intronic regions of human PRPH2. Minigenes carrying wild type PRPH2 or PRPH2 exon 2 mutations associated with rod or cone disorders were expressed in murine photoreceptors using recombinant adeno-associated virus (rAAV) vectors. We detect three PRPH2 splice isoforms in rods and cones: correctly spliced, intron 1 retention, and unspliced. In addition, we show that only the correctly spliced isoform results in detectable protein expression. Surprisingly, compared to rods, differential splicing leads to lower expression of correctly spliced and higher expression of unspliced PRPH2 in cones. These results were confirmed in qRT-PCR experiments from FAC-sorted murine rods and cones. Strikingly, three out of five cone disease-causing PRPH2 mutations profoundly enhanced correct splicing of PRPH2, which correlated with strong upregulation of mutant PRPH2 protein expression in cones. By contrast, four out of six PRPH2 mutants associated with rod disorders gave rise to a reduced PRPH2 protein expression via different mechanisms. These mechanisms include aberrant mRNA splicing, protein mislocalization, and protein degradation. Our data suggest that upregulation of PRPH2 levels in combination with defects in the PRPH2 function caused by the mutation might be an important mechanism leading to cone degeneration. By contrast, the pathology of rod-specific PRPH2 mutations is rather characterized by PRPH2 downregulation and impaired protein localization.

## Author Summary

Photoreceptors are the light sensing cells of the retina and consist of dim light and night vision mediating rods and daylight and color vision mediating cones. PRPH2 is crucial for the structural and functional integrity of photoreceptors. Some point mutations in PRPH2 lead to degeneration of rods, whereas others only affect cones. We examined the potential effects of 11 disease-linked PRPH2 mutations on mRNA splicing and protein expression *in vivo*. For this, we expressed six PRPH2 mutants associated with degeneration of rods in murine rods and five additional mutants linked to cone diseases in murine cones. We demonstrate that different splicing efficiencies of PRPH2 lead to its high expression in rods and to its low expression in cones. Furthermore, we show that the majority of PRPH2 mutants associated with cone disorders results in an upregulation of PRPH2 expression in cones by increasing the mRNA splicing efficiency. By contrast, the majority of PRPH2 mutants associated with rod diseases leads to a downregulation of PRPH2 expression in rods via different mechanisms including aberrant mRNA splicing. These results provide novel insights into the pathobiology of mRNA splicing in photoreceptors and might contribute to explain the differential penetrance of PRPH2 mutants in rods and cones.

## Introduction

The retina converts light into electrical signals via two highly specialized types of neurons, rod and cone photoreceptors. Both types of photoreceptors express in their light-sensitive outer segments (rod outer segments, ROS and cone outer segments, COS, respectively) a specific member of the tetraspanin protein family, peripherin-2 (PRPH2). PRPH2 is a glycosylated protein harboring four transmembrane helices and was found to be crucial for development and structural integrity of photoreceptor outer segments [1, 2]. Mutations in the human PRPH2 gene are linked to retinal disorders affecting the structure or function of rods or cones. Some of these mutations lead to autosomal dominant retinitis pigmentosa (adRP), a degenerative disease that primarily affects the rod photoreceptors, whereas others result in different types of autosomal dominant disorders characterized by cone defects [3]. The molecular pathways underlying the rod *vs* cone specificity of individual PRPH2 mutations are currently unknown. The vast majority of PRPH2 mutations are point mutations and many of them are located in exon 2, which encodes for the distal half of the loop domain connecting the third and fourth transmembrane segment (D2 loop), and the proximal half of the fourth transmembrane domain. Previous studies demonstrated that disease-associated exonic point mutations can affect regulatory elements of mRNA splicing via different mechanisms [4]. These mechanisms include the abolition of existing or generation of novel donor or acceptor sites [4, 5]. Alternatively, exonic point mutations may also affect the binding motifs of so-called exonic splice enhancers (ESEs) or exonic splice silencers (ESSs) [6, 7]. These binding motifs usually comprise 4–18 nucleotides and regulate constitutive and alternative splicing [8, 9]. Point mutations that alter existing or generate novel binding sites for ESEs or ESSs can either lead to skipping of the respective exon or to the retention of flanking introns [4, 8, 10]. Moreover, differential splicing occurs in a highly cell-specific manner [11] highlighting the need to analyze splicing defects in the cell type of interest (e.g. rod or cone photoreceptor). A high number of disease-linked exonic point mutations have been identified in genes known to be crucial for the functional and structural identity of photoreceptors (https://sph.uth.edu/retnet/disease.htm). However, the potential effects of these point mutations on mRNA splicing in their native environment have not been examined for any of these genes so far. Moreover, studies addressing the effects of exonic point mutations on regulatory splicing elements were performed on minigenes that do not cover the full-length coding sequence and, hence, do not permit concomitant analysis of how the mutations affect protein expression [12]. However, side-by-side analysis of protein expression is essential to dissect the mechanisms leading to diseased states. To examine the impact of exonic PRPH2 point mutations on splicing and protein expression in photoreceptors, we constructed rAAV vectors that express PRPH2 minigenes containing native canonical splice sites and all three coding exons of PRPH2 under control of either a rod or a cone specific promoter. Using this approach, we unravel cell type- and mutation-specific differential mRNA splicing as a novel mechanism regulating PRPH2 protein expression in healthy and diseased rod and cone photoreceptors.

## Results

### rAAV-PRPH2 minigenes confer efficient expression of PRPH2 in rods and cones

**Fig 1:**
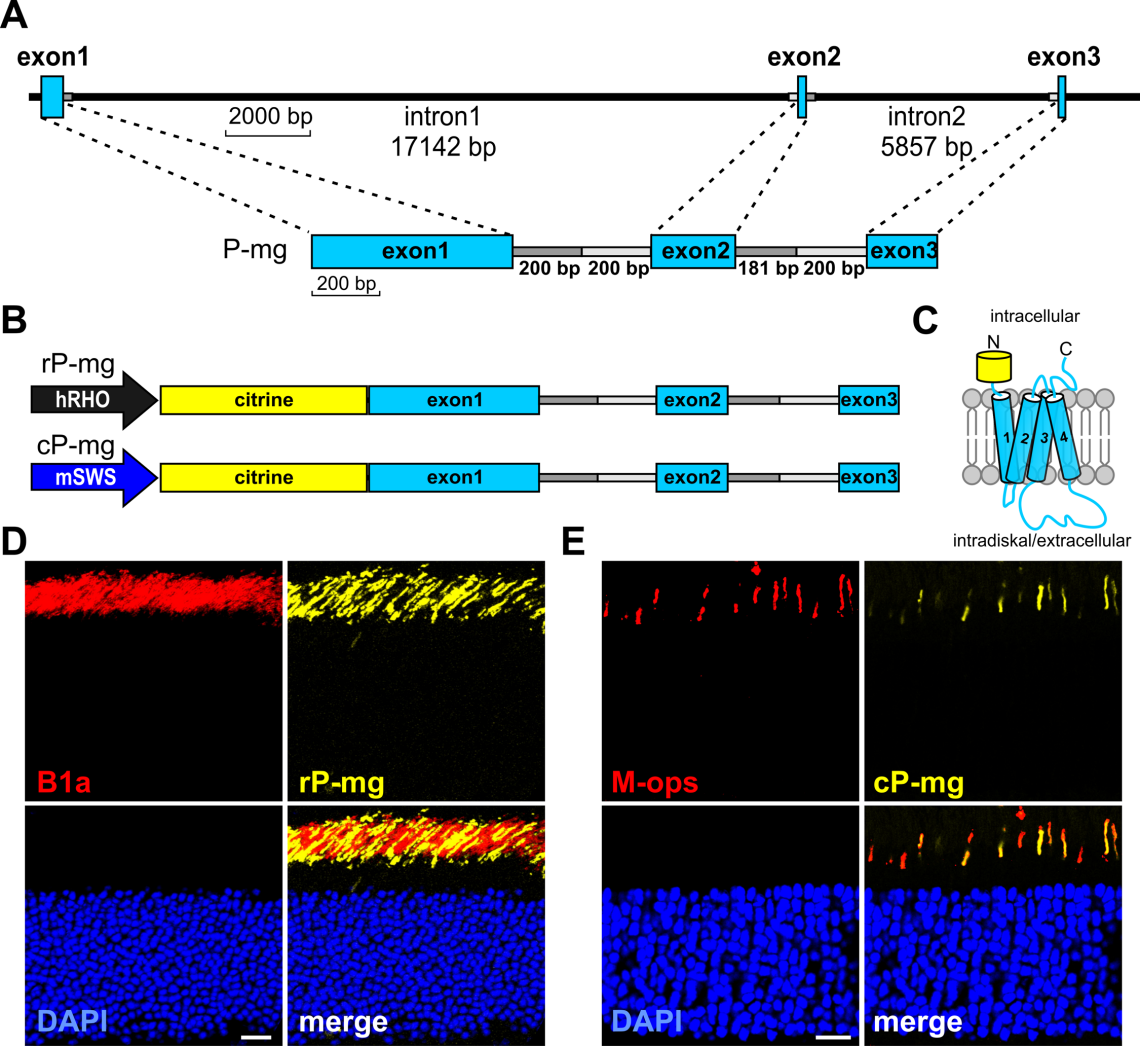
Design and *in vivo* expression of PRPH2 minigenes. (A) Exon-intron structure of native human PRPH2 (upper panel) and derived PRPH2 minigene used in this study (lower panel). In the minigene, intron 1 and intron 2 were largely deleted with the exception of sequences flanking exons 1–3 as indicated. (B) PRPH2 minigene constructs containing different promoters used for the analysis in rods (rP-mg; top), and cones (cP-mg; bottom). hRHO, human rhodopsin promoter; mSWS, murine S-opsin promoter. To allow antibody-free visualization of minigene-derived PRPH2, a citrine tag was fused to exon 1 (N-terminus) of PRPH2. (C) Topology of the correctly spliced citrine-tagged PRPH2. N- and C-termini face the intracellular side of photoreceptors. By contrast, the D2 loop is located on the intradiskal (rods) or extracellular (cones) side (cf. S1 Fig). (D and E) Immunohistology of murine retinas injected on P14 with rP-mg (D) and cP-mg (E), respectively. Retinas were harvested three weeks post injection. Scale bar represents 20 μm. CNGB1a (B1a) and M-opsin (M-ops) antibodies were employed to label rod and cone outer segments, respectively.

The entire human PRPH2 gene including the 5’ and 3’ untranslated region, the exons, and the introns encompasses approximately 26 kb (Fig 1A, upper panel). Consequently, with regard to the limited capacity of the rAAV vectors [13], splicing cannot be analyzed on the native PRPH2 transcript. In an attempt to study PRPH2 splicing in rod and cone photoreceptors, we deleted most of the intronic sequences with the exception of 180–200 bp flanking the exons and the 5’ end of the PRPH2 gene (Fig 1A, lower panel). This construct was fused to a citrine tag to allow monitoring of minigene-derived PRPH2 expression. The resulting construct was inserted in the rAAV vector containing either a human rhodopsin (i.e. rod-specific) or murine short wavelength opsin (i.e. cone-specific) promoter yielding minigene vectors rP-mg and cP-mg, respectively (Fig 1B). Correct splicing of these minigenes leads to an N-terminally citrine-tagged PRPH2 with a molecular weight of approx. 66.5 kDa (Fig 1C). rP-mg and cP-mg were packaged as AAV2/8 Y733F viral particles [13, 14] and delivered into the subretinal space of two week old (P14) wild type mice. Subsequent analysis of minigene-derived PRPH2 expression was monitored three weeks post injection by citrine fluorescence on retinal slices from injected animals. In both, ROS and COS, citrine was exclusively expressed in the outer segments (Fig 1D and 1E).

### *In silico* mRNA splice analysis of exon 2-specific PRPH2 point mutations

Exon 2 of PRPH2 represents a mutational hotspot and encodes for the distal part of the D2 loop domain and for the proximal half of transmembrane domain 4 (S1 Fig). Initially, we performed *in silico* prediction of the potential effects of 30 exon 2-specific PRPH2 point mutations on aberrant mRNA splicing using the ASSEDA and NNSplice splice prediction software. For *in silico* prediction, we used 40 bp sequences flanking the respective mutation from each site. This analysis provided a variety of predicted effects on mRNA splicing for many mutants including the potential generation of novel donor or acceptor sites and the abolition or generation of binding sites for ESEs or ESSs (S1 Table). To test the potential differences of PRPH2 mutants on mRNA splicing experimentally, we randomly chose eleven point mutants predicted to impact splicing from the *in silico* analysis. Six of these mutants are associated with autosomal-dominant retinitis pigmentosa (adRP), the most common type of inherited autosomal-dominant degenerative retinal disorders, whereas five are found in patients suffering from different types of cone diseases (Table 1). None of these mutants have been previously analyzed for their potential effects on mRNA splicing.

**Table 1:** Point mutations in exon 2 of PRPH2 analyzed in this study [15–25].

Mutation	Disease	References
*c*.*584G>T; p*.*R195L*	cone and cone-rod dystrophy	Yanagihashi *et al*. 2003
*c*.*594C>G; p*.*S198R*	adRP	Sullivan *et al*. 2006
*c*.*625G>A; p*.*V209I*	adult foveomacular vitelliform dystrophy	Coco *et al*. 2010
*c*.*629C>T; p*.*P210L*	adRP	Budu *et al*. 2001
*c*.*635G>C; p*.*S212T*	adult foveomacular vitelliform dystrophy	Felbor *et al*. 1997
*c*.*641G>C; p*.*C214S*	adRP	Saga *et al*. 1993
*c*.*658C>T; p*.*R220W*	pattern dystrophy	Payne *et al*. 1998
*c*.*659G>A; p*.*R220Q*	pattern dystrophy	Jacobson *et al*. 1996
*c*.*676C>G; p*.*Q226E*	adRP	Rodriguez *et al*. 1994
*c*.*736T>C; p*.*W246R*	adRP	Kohl *et al*. 1997
*c*.*745G>A; p*.*G249S*	adRP	Renner *et al*. 2009

Six mutations lead to autosomal dominant retinitis pigmentosa (adRP) and five mutations are linked to cone diseases.

### Comparative splice analysis of wild type and mutant PRPH2 minigenes in rods and cones

**Fig 2:**
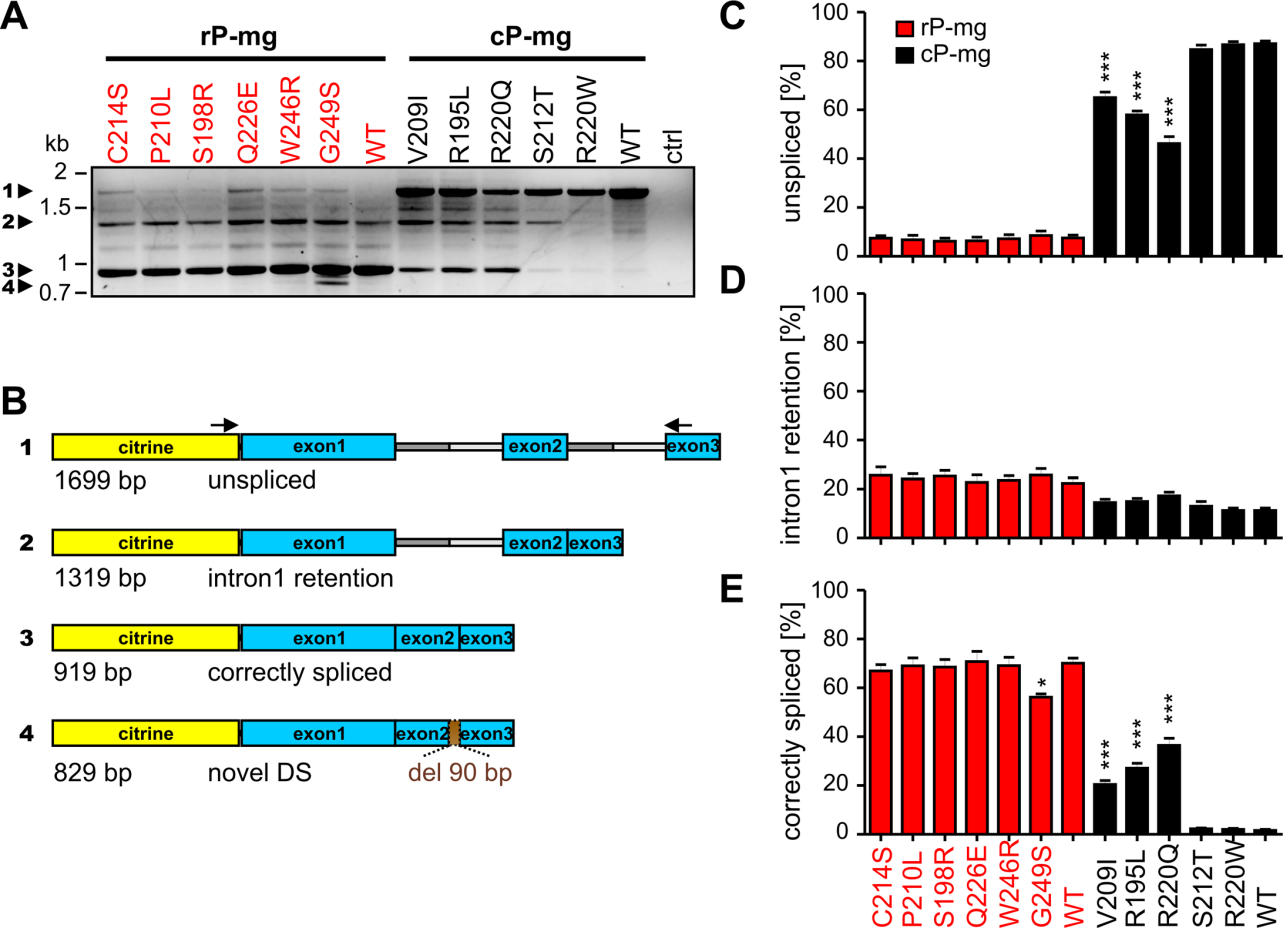
Splice analysis of PRPH2 WT and mutant minigenes in rods and cones. (A) Representative RT-PCR from cDNA generated from total RNA three weeks post injection from retinas injected with wild-type and mutant rP-mg (left) or cP-mg (right) on P14. Ctrl, control containing the cDNA from non-transduced retina. The single bands of the relevant splice products are numbered (1–4) and highlighted by arrowheads. (B) Schematic representation of the detected splice variants using primers binding to the 3’-end of citrine and to the 5’-end of exon 3 as indicated by the arrows. The numbers of the constructs correspond to the bands marked in (A). (C-E) Semi-quantitative analysis of the relative intensities of the unspliced (C), intron 1 retention (D), and correctly spliced (E) PRPH2 transcripts. For each PRPH2 minigene, the mean percentage of the intensities of these three variants relative to the total intensity (given as sum of the single intensities) was calculated from five RT-PCR analyses conducted with a variable number of cycles (25–27 for rods and 30–32 for cones, respectively). Significance test of the rod or cone mutants to the corresponding wild type (one-way ANOVA followed by Dunett’s test) was performed for rP-mg and cP-mg, respectively. All data were shown as mean values and the error bars represent the standard error of the mean (SEM). *, p< 0.05; **, p< 0.01; ***, p< 0.001. DS, splice donor site.

We analyzed the mRNA splicing of the PRPH2 point mutants using RT-PCR performed with minigene-specific primers and compared the pattern of the splice products after rod- or cone-specific expression, respectively (Fig 2A). For this purpose, wild type (WT) mice were injected at P14 with WT or respective mutant PRPH2 constructs. Three weeks after injection, RNA was isolated and pooled from four retinas of four injected animals. The identity of transcripts was confirmed by direct sequencing of the purified amplicons (S2 Fig). Three different splice products were obtained for WT and mutant PRPH2 minigenes in rods and cones. These three splice products corresponded to i) the unspliced variant containing both introns, ii) the splice variant with retained intron 1, and iii) the correctly spliced PRPH2. The adRP-associated *G249S* mutation gave rise to a fourth splice variant that contained an in-frame deletion of 90 bp at the 3’ end of exon 2 caused by the use of a novel donor site (DS) (Fig 2A and 2B). However, the most intriguing finding was that, other than in rods, the unspliced PRPH2 transcript was the most prominent splice isoform in cones and the correctly spliced PRPH2 transcript was present at lower amounts in this cell type (Fig 2A).

### Semi-quantitative analysis of the PRPH2 splice isoforms

For quantification of the three PRPH2 splice variants (unspliced, intron 1 retention, and correctly spliced), we injected mice at P14 with rAAVs expressing WT or mutant PRPH2 minigenes and isolated the RNA three weeks post injection. For RT-PCR experiments, we used RNA isolated from a set of four injected retinas pooled for each WT or mutant construct. To determine the linear range of amplification, we performed quantitative RT-PCR (qRT-PCR) from mice injected with WT PRPH2 in rods and cones using primers as indicated in Fig 2B. Linear range of amplification was between 20–28 cycles for rods and between 26–33 cycles for cones, respectively (S3 Fig). Subsequently, five technical RT-PCR replicates were conducted using a variable number of cycles falling within the respective range of linear amplification in rods and cones. We calculated the mean percentage of unspliced, intron 1 retention, and the correctly spliced transcripts relative to the sum of band intensities for all PRPH2 minigenes. The data analysis revealed profound differences in the relative percentage of the single splice isoforms in rods and cones, respectively. These differences were observed when comparing the splicing of WT PRPH2 minigenes in rods and cones, but also when relating the effects of the single mutants to the corresponding WT in a given cell type. For WT PRPH2 in rods, the major splice isoform was the correctly spliced transcript (approx. 70%), whereas splicing of WT PRPH2 in cones yielded only low amounts of this PRPH2 isoform (approx. 2%, Fig 2E and Table 2). By contrast, in cones, splicing of WT PRPH2 predominantly resulted in the unspliced isoform (87%, Fig 2C). A moderate difference between WT PRPH2 in rods and cones could be observed for the intron 1 retention isoform (22% in rods *vs* 11% in cones, Fig 2D). Next, we compared the relative percentages of splice bands for single PRPH2 mutants to the respective WT expressed in rods and cones. No variability in the percentage of the intron 1 retention isoform was observed for any of the mutants in rods and cones (Fig 2D). However, the amount of the correctly spliced isoform was reduced to 51% for the *G249S* rod-dominant mutation (Fig 2E and Table 2). This reduction most likely results from the generation of the novel donor site observed for this mutant. By contrast, in cones, three mutants (*V209I*, *R195L*, and *R220Q*) resulted in a strong increase in the percentage of the correctly spliced PRPH2 isoform, which was accompanied by an appropriate decrease in the unspliced transcript (Fig 2C and 2E and Table 2). Taken together, these results indicate cell-type and mutation specific effects on mRNA splicing of PRPH2 in rods and cones.

**Table 2:** Relative intensity percentages of PRPH2 isoforms for WT and mutant PRPH2 minigenes in rods and cones.

		C214S	P210L	S198R	Q226E	W246R	G249S	WT	V209I	R195L	R220Q	S212T	R220W	WT
**unspliced**	**MV**	7.48	6.79	6.17	6.41	7.22	8.53	7.54	65.01	57.95	46.21	84.74	86.68	87.09
	**SEM**	1.00	1.85	1.25	1.43	1.70	1.84	1.19	2.25	1.60	2.86	1.78	1.24	1.12
	**Dunett's test**	n.s.	n.s.	n.s.	n.s.	n.s.	n.s.		n.s.	n.s.	n.s.	n.s.	n.s.	
**intron 1 retention**	**MV**	25.60	24.15	25.25	22.81	23.66	25.73	22.35	14.52	14.94	17.33	13.00	11.31	11.27
	**SEM**	3.40	2.15	2.38	2.99	1.77	2.64	2.26	1.38	1.26	1.50	1.97	0.95	1.06
	**Dunett's test**	n.s.	n.s.	n.s.	n.s.	n.s.	n.s.		n.s.	n.s.	n.s.	n.s.	n.s.	
**correctly spliced**	**MV**	66.92	69.05	68.57	70.78	69.13	56.19	70.11	20.47	27.10	36.46	2.25	2.01	1.64
	**SEM**	2.65	3.21	3.09	4.16	3.39	1.31	2.09	1.59	2.04	2.89	0.39	0.43	0.42
	**Dunett's test**	n.s.	n.s.	n.s.	n.s.	n.s.	p<0.05		p<0.0001	p<0.0001	p<0.0001	n.s.	n.s.	

MV, mean value

SEM, standard error of the mean.

n.s., not significant.

### Quantitative analysis of native PRPH2 splice isoforms

**Fig 3:**
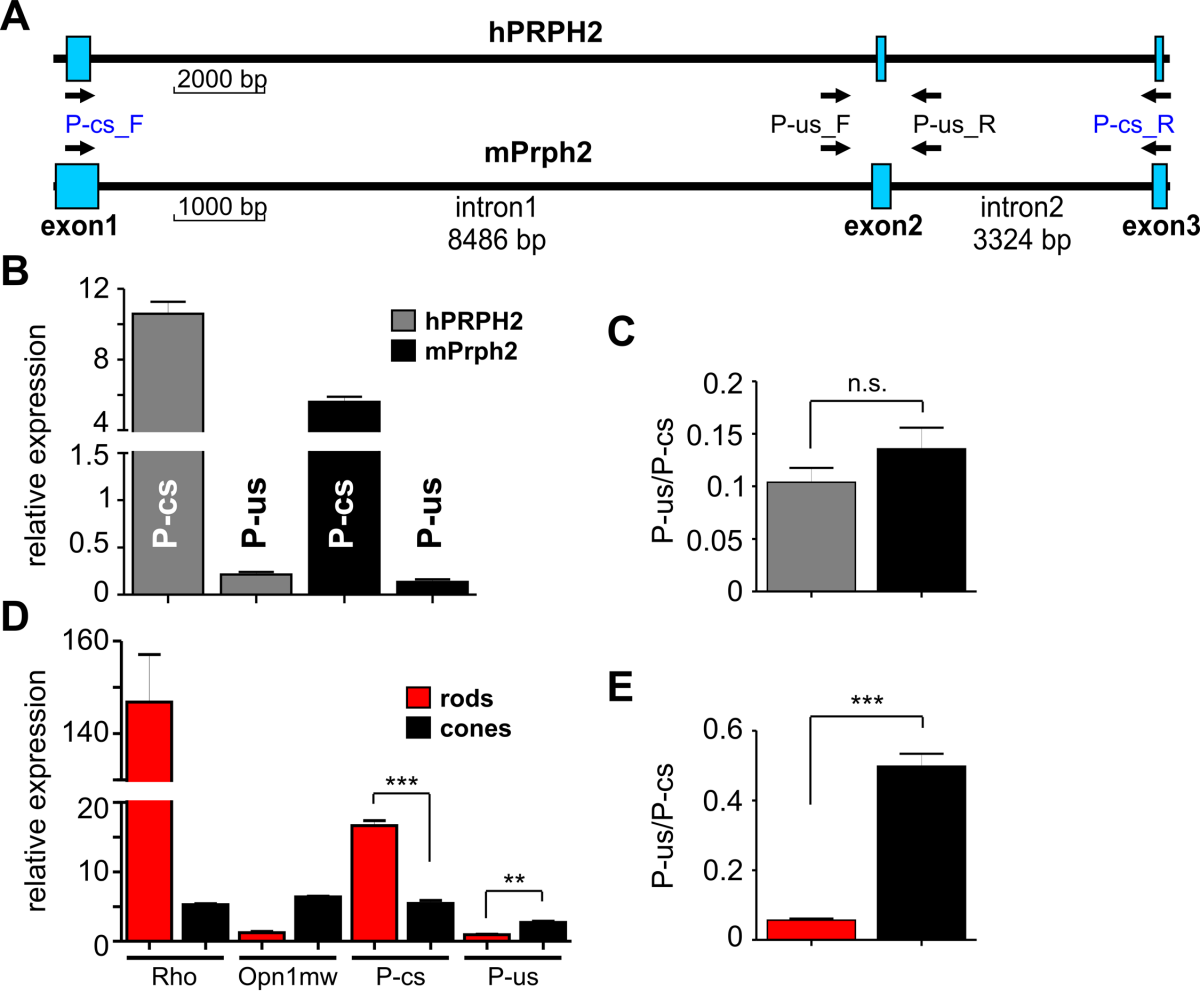
Quantification of PRPH2 splice isoforms in native human and murine photoreceptors. (A) True-to-scale representation of the exonic and intronic regions of human PRPH2 (upper panel) and mouse Prph2 (lower panel). The binding positions of primers used for qRT-PCR are indicated by arrows. For correctly spliced human PRPH2 or murine Prph2 (hP-cs or mP-cs), the P-cs_F and P-cs_R primer combination was used. For detection of the unspliced human (hP-us) or murine (mP-us) variant, the P-us_F and P-us_R primer combination was applied. (B) qRT-PCR from pooled human (gray boxes) or mouse (black boxes) total retinal RNA isolated from two (human) or four (mouse) biological samples. The single values are as follows: hP-cs, 10.60 ± 0.67; hP-us, 0.21 ± 0.03; mP-cs, 5.60 ± 0.29; mP-us, 0.13 ± 0.03. All data are given as mean values and error bars represent the SEM. Three technical replicates were conducted for each primer combination and the expression was normalized to the housekeeper aminolevulinic acid synthase (ALAS). (C) Relative ratios given as mean values ± SEM of the single unspliced transcripts to the corresponding correctly spliced isoform from human (0.10 ± 0.01) or mouse (0.13 ± 0.02) retina. Significance test was performed with the two-tailed t-test (p = 0.26). (D) qRT-PCR analysis from sorted murine rods (red boxes) and cones (black boxes), respectively. For qRT-PCR, cDNA resulting from pooled rods sorted from six animals (yielding 100.000 cells) and pooled cones sorted from four animals (yielding 27.000 cells) were used. cDNA synthesis was performed using identical total RNA concentration for rods and cones, respectively (50 ng each). For purity assessment of FAC-sorted rods and cones, primers specific for murine rhodopsin (*Rho*) and M-opsin (*Opn1mw*) were used. For detection of Prph2 splice isoforms, the same primer combinations were applied as indicated in Fig 3A. Three technical replicates were conducted for each primer combination and the expression was normalized to the housekeeper aminolevulinic acid synthase (Alas). All data are given as mean values and error bars represent the SEM. The single values for FAC-sorted rods are as follows: Rho, 146.90 ± 10.24; Opn1mw, 1.25 ± 0.16; P-cs, 16.67 ± 0.71; P-us, ± 0.95 ± 0.07. Expression in sorted cones yielded following values: Rho, 5.27 ± 0.14; Opn1mw, 6.38 ± 0.13; P-cs, 5.45 ± 0.44; P-us, ± 2.71 ± 0.19. P-cs rods *vs* cones: p = 0.0002; P-us rods *vs* cones: p = 0.001. (E) Relative ratios of the single unspliced transcripts to the corresponding correctly spliced isoform from FAC-sorted rods or cones. Significance analysis was performed with the two-tailed t-test (p = 0.0003).

The experiments shown in Fig 2A were performed using minigenes that contain human PRPH2 expressed in murine photoreceptors. However, it is conceivable that endogenous abundance of the single PRPH2 splice isoforms might differ in human and in mouse photoreceptors, which in turn would impede the interpretation of our minigene-based assay. To examine if murine photoreceptors are suitable for the splicing analysis of human PRPH2, we investigated the endogenous levels of PRPH2 splice isoforms in human and murine retina using qRT-PCR. To this end, we utilized two specific primer sets amplifying the two human or murine PRPH2 transcripts mirroring the splice isoforms obtained in the minigene experiments (Fig 3A). Specific amplification of the unspliced PRPH2 transcript was assessed using primers binding to the intronic regions flanking exon 2 (P-us_F and P-us_R). To detect the correctly spliced PRPH2 isoform, we used primers binding to exon 1 and exon 3 (P-cs_F and P-cs_R). Due to the large size of the interjacent native introns (23 kb for human and 12 kb for murine intron 1 and intron 2, respectively, cf. Fig 1A and Fig 3A), PRPH2 isoforms bearing the introns are most likely not amplified under the qRT-PCR conditions. For quantification, the relative expression of all single isoforms was normalized to the endogenous housekeeper aminolevulinic acid synthase (ALAS). Importantly, both primer combinations led to clearly detectable qPCR products in both, human and mouse retina with the relative PRPH2 expression being slightly higher for both combinations in the human retina (Fig 3B). Of note, given the fact that, in human and mouse retina, rods account for >95% of the photoreceptor population, the expected results should reflect the splicing of PRPH2 minigenes in rods. In line with this, we found the correctly spliced PRPH2 isoform to be much higher expressed in native retina when compared to the unspliced variant (10.60 ± 0.68 *vs* 0.21 ± 0.03 for human, and 5.60 ± 0.29 *vs* 0.14 ± 0.03 for murine retina, respectively). No differences between human and mouse PRPH2 expression could be detected when plotting the ratio of the unspliced to the corresponding correctly spliced PRPH2 isoforms (0.10 ± 0.01 for human *vs* 0.13 ± 0.02 for murine retina, respectively (Fig 3C)). Taken together, these results strongly indicate that in both, qualitative and quantitative terms, splicing of PRPH2 is very similar in the human and the mouse retina.

Our PRPH2 minigene results revealed that cones significantly differ from rods in relative abundance of the correctly spliced and the unspliced PRPH2 variants (Fig 2A). To test if this also holds true in the native context, we purified rods and cones from 6–8 weeks old mice by fluorescence-activated cell sorting (FACS) and subsequently determined the abundance of endogenous murine Prph2 splice isoforms in sorted photoreceptors [26, 27]. The purity of sorted photoreceptors was validated by assessing the transcript levels of the cell type-specific genes rhodopsin (*Rho*) and M-opsin (*Opn1mw*) as markers for rods and cones, respectively. Compared to the FAC-sorted rods, sorted cones displayed a 28-fold reduction of Rho expression accompanied by a 5-fold increase of M-opsin expression (Fig 3D). This indicates a high purity of sorted cones showing only a slight contamination with rods (about 3.6%). In line with the qRT-PCR results shown in Fig 3B for the native retina, in FAC-sorted rods, we found the correctly spliced Prph2 isoform to be much higher expressed when compared to the unspliced variant (16.67 ± 0.71 *vs* 0.95 ± 0.07, Fig 3D). By contrast, in FAC-sorted cones, the expression of correctly spliced Prph2 displayed only a very moderate increase compared to the unspliced variant (5.45 ± 0.44 *vs* 2.71 ± 0.19). Concomitantly, these data also demonstrate that the relative expression of correctly spliced Prph2 was decreased in cones, which was accompanied by an increase in the expression of the unspliced Prph2 transcript. These differences in expression of the single Prph2 isoforms between rods and cones become even more obvious when comparing the ratios of the unspliced to the correctly spliced Prph2 isoforms between the two cell types (0.06 ± 0.004 for rods and 0.49 ± 0.04 for cones, respectively (Fig 3E)). Taken together, these data are largely compatible with the data observed from our minigenes and show that the relative endogenous levels of correctly spliced and unspliced Prph2 isoforms differ significantly between rods and cones.

### PRPH2 isoform encoded by exon 1 is mislocalized in photoreceptors

**Fig 4:**
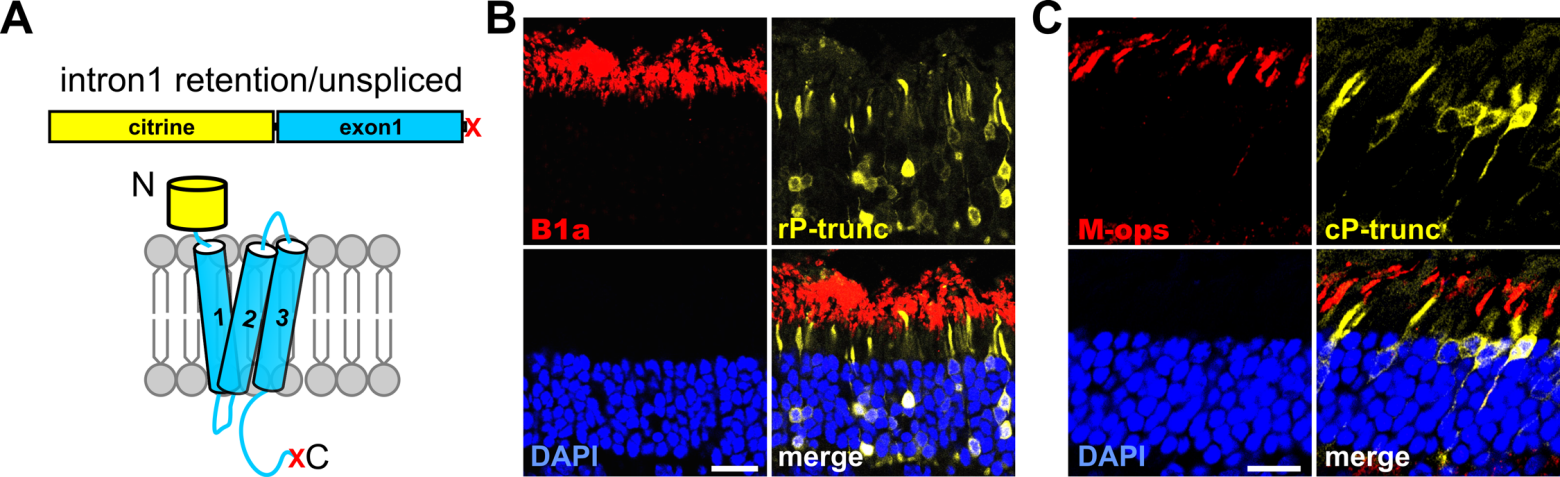
Impaired targeting of truncated PRPH2. Immunohistology of transduced murine retinas showing rod- (B) and cone-specific (C) expression of a truncated version of PRPH2. (A) Truncated PRPH2 contains only exon 1 and a downstream stop codon (indicated by “X”) mimicking translation from unspliced PRPH2 mRNA and PRPH2 mRNA with intron 1 retention. Staining for B1a and M-ops was used to label rod and cone photoreceptors, respectively. Truncated PRPH2 is not transported to outer segments and is almost exclusively present in inner segments and somata of photoreceptors. Scale bar represents 20 μm.

Results shown in Fig 1D and 1E indicate that minigene-derived, fluorophore-tagged PRPH2 protein is correctly expressed and localized in murine rod and cone outer segments. However, since all minigene-derived PRPH2 splice isoforms bear the fluorophore, the detection of the fluorophore signal does not allow to discriminate which of these isoforms are expressed on protein level. The correctly spliced, fluorophore-tagged PRPH2 leads to the full-length protein known to be properly localized in the outer segments [28]. However, the remaining two PRPH2 splice isoforms (unspliced and intron 1 retention) harbor a premature stop codon immediately after exon 1 and their expression was not analyzed in previous studies. Translation of these two PRPH2 transcripts would result in a truncated PRPH2 protein lacking the distal half of the D2 loop, TM4, and downstream sequence (Fig 4A and S2 Fig panel C). With respect to the translation and protein expression of these PRPH2 isoforms, one can in principle assume four different possibilities: i) The two splice isoforms are translated into protein and the protein is stable, but mislocalized to the inner segments. ii) The two splice isoforms are translated into protein and the protein is correctly localized. iii) They are translated into protein, but the protein is degraded. iv) They are not translated into protein, but are degraded on mRNA level, i.e. via the nonsense-mediated mRNA decay (NMD) mechanism, which requires the presence of a premature stop codon within the mRNA. The first possibility can be excluded since we do not observe any mislocalized protein in transduced photoreceptors upon expression of the WT PRPH2 minigene. To discriminate between the remaining three scenarios we expressed the PRPH2 variant that harbors a stop codon immediately after exon 1 and, therefore, mirrors the protein that is translated from the unspliced or intron 1 retention transcript (cf. S2 Fig panel C). However, in contrast to the two minigene-derived PRPH2 splice isoforms, this variant does not contain a premature stop codon and, thus, the mRNA cannot be degraded via the NMD mechanism. Expression of the truncated PRPH2 in rod or cone photoreceptors leads to a protein that is highly expressed, but is completely mislocalized to the inner segments (Fig 4B and 4C). Thus, scenario ii) or iii) proposing correctly localization or protein degradation of this PRPH2 isoform could be excluded. Based on these findings, we conclude that the two splice isoforms harboring a premature stop codon are not translated into protein, presumably due to mRNA degradation via the NMD mechanism.

### Protein expression and localization of PRPH2 mutants in rods and cones

**Fig 5:**
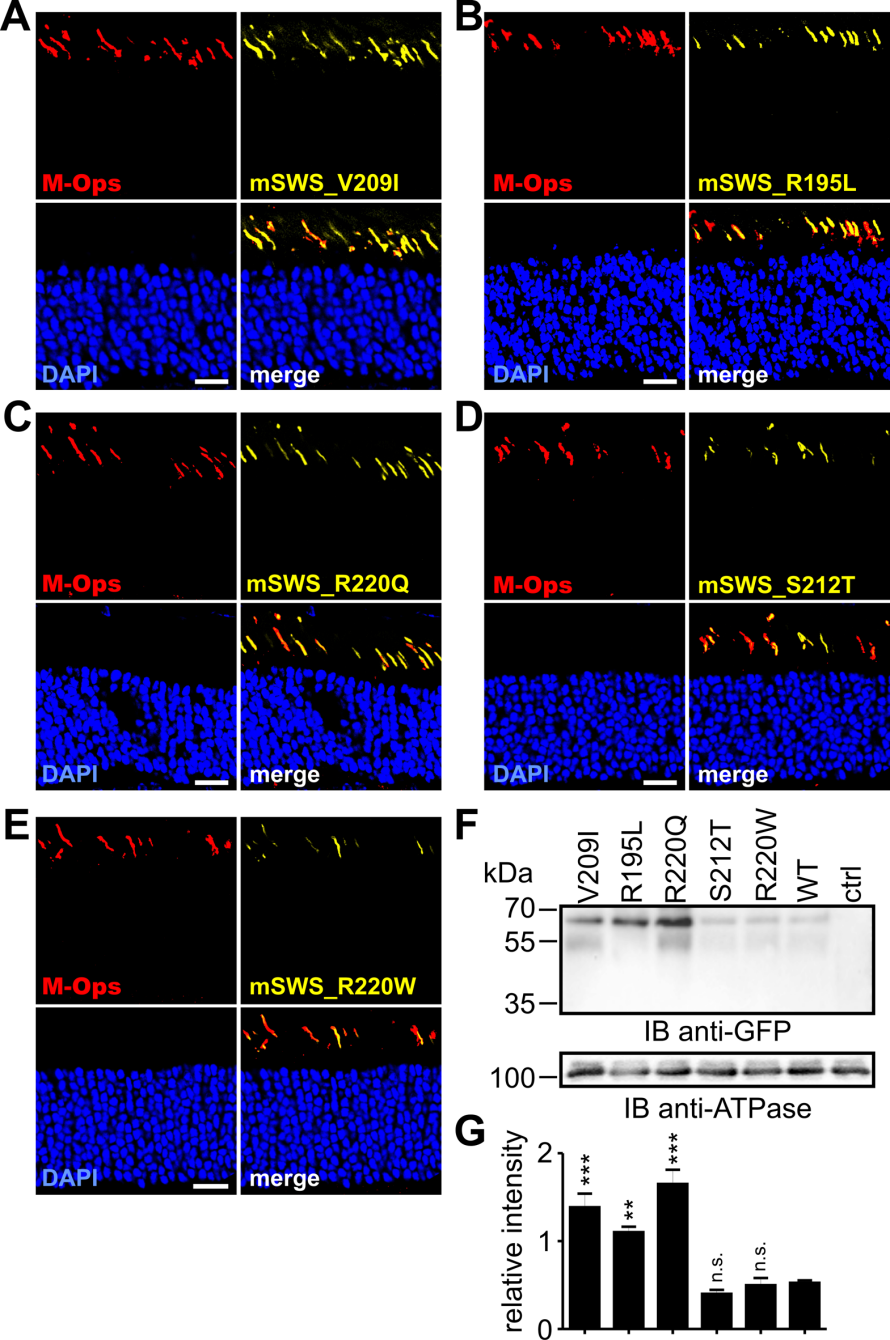
Retinal expression of PRPH2 mutants linked to cone diseases. (A-E) Immunohistological analysis of retinas transduced with the PRPH2 minigenes carrying single point mutations under the control of the mSWS promoter as indicated. Scale bar represents 20 μm. (F) Western blot analysis from membrane preparations of murine retinas transduced with the PRPH2 minigenes shown in A-E. Western blotting was conducted using four pooled retinas from four animals injected on P14. All retinas were collected three weeks post injection. Ctrl, protein lysates from non-injected control retinas. PRPH2 was detected by an anti-GFP antibody that recognized the citrine tag. As loading control, an antibody against the murine alpha subunit of ATPase was used (anti-ATPase). (G) Semi-quantitative analysis of the results shown in (F). For quantification, three technical replicates were conducted and PRPH2 expression was normalized to the ATPase expression. All data are given as mean values ± SEM. Statistical analysis was performed using one-way ANOVA followed by the Dunett’s test. *, p< 0.05; **, p< 0.01; ***, p< 0.001. n.s., not significant.

**Fig 6:**
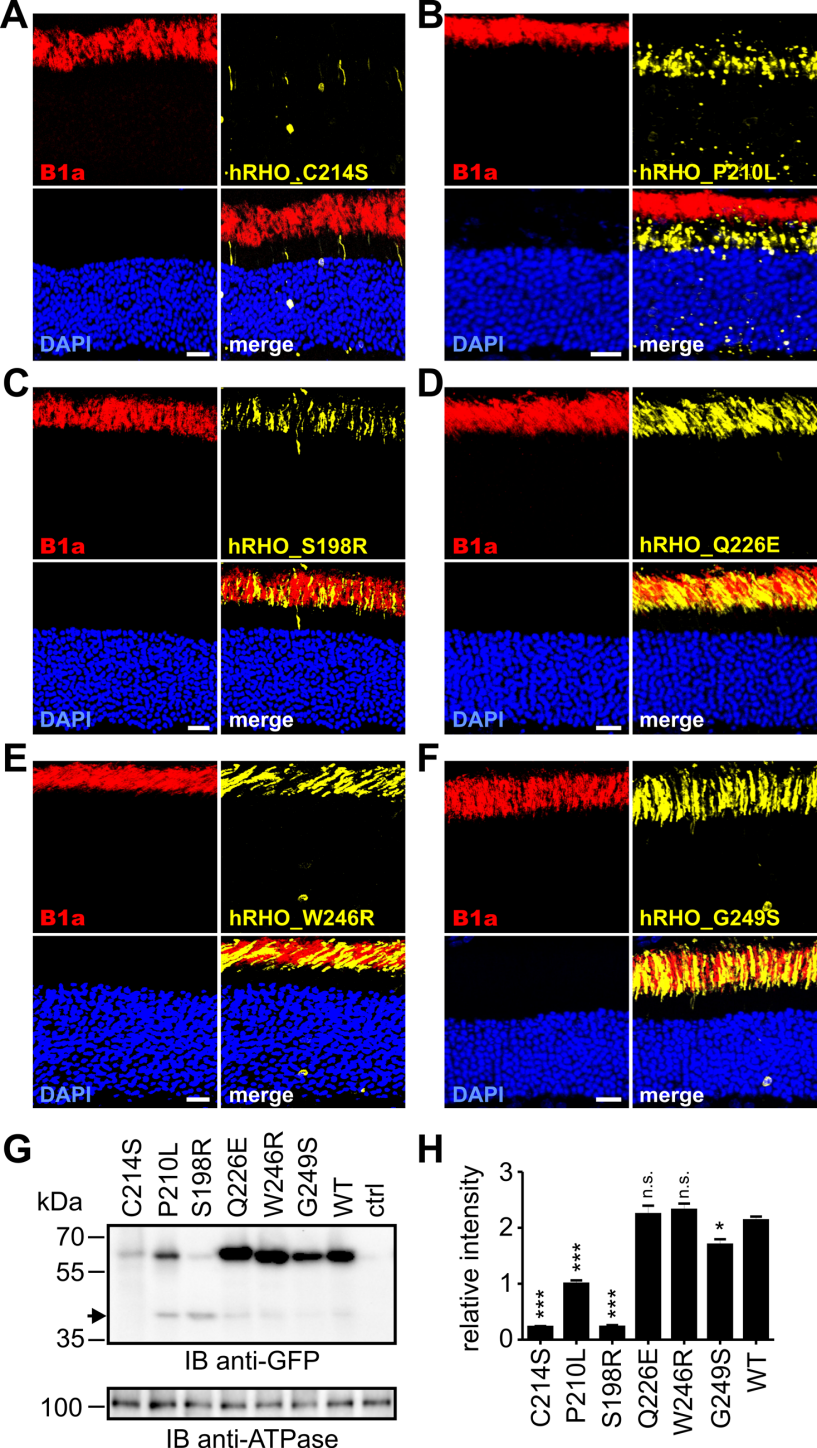
Protein expression of PRPH2 mutants linked to adRP. (A-F) Immunohistology of retinas transduced with PRPH2 minigenes containing single point mutations as indicated under the control of the hRHO promoter. Scale bar represents 20 μm. (G) Western blot analysis from membrane preparations of four pooled murine retinas from four animals transduced with the PRPH2 minigenes shown in (A-F) on P14. All retinas were collected three weeks post injection. The arrowhead indicates a degradation band detected at 42 kDa. Ctrl, protein lysates from non-injected control retinas. (H) Semi-quantitative analysis of the results shown in (G). For quantification, three technical replicates were conducted and PRPH2 expression was normalized to the ATPase expression. All data are given as mean values and error bars represent the SEM. Statistical analysis was performed using one-way ANOVA followed by the Dunett’s test. *, p< 0.05; **, p< 0.01; ***, p< 0.001. n.s., not significant.

We next used PRPH2 minigene rAAV vectors to analyze the protein expression and retinal localization of PRPH2 mutants linked to cone diseases (Fig 5). All five mutants produced significant levels of proteins that were correctly localized to the cone outer segments (Fig 5A–5E). In Western blot analysis, the mutant proteins migrated at the size of the wild type full-length PRPH2 (approx. 66.5 kDa), however, expression levels differed between the various mutant and the wild type protein (Fig 5F). In particular, we detected a robust increase in protein expression for the *V209I*, *R195L*, and *R220Q* mutants compared to the WT PRPH2 (Fig 5F and 5G). These findings are in line with the strongly increased splicing efficiency of these three mutants resulting in higher levels of correctly spliced PRPH2 in cones (cf. Fig 2). In contrast to uniform behavior of "cone-specific" PRPH2 mutations, "rod-specific" PRPH2 mutants linked to adRP revealed more diverse effects. In particular, for the *C214S* mutant, whose effect on protein localization was also previously investigated [29], we confirmed a severe protein mislocalization to the inner segment and throughout the photoreceptor cell (Fig 6A). Similar to *C214S*, we found that also the *P210L* mutant results in massive mislocalization to the inner segment (Fig 6B). However, in contrast to the *C214S* mutant that showed an evenly cytosolic distribution pattern, the *P210L* mutant was found in large and speckled vesicular-like structures. In addition to these findings, the Western blot analysis revealed a more or less pronounced reduction of protein expression for four adRP-linked PRPH2 mutations (Fig 6G and 6H). The strongest reduction was observed for the *C214S* and for the *S198R* mutant, followed by the *P210L* and the *G249S* mutation. By contrast, the other two analyzed mutants resulted in wild type-like protein levels (Fig 6G and 6H). Interestingly, two of the mutants (*S198R* and *P210L*) displayed an additional approx. 42 kDa band on the Western blot most likely resulting from protein degradation. Such degradation was described for mutations causing protein misfolding, which could lead to the accessibility for a specific protease cleavage site that is hidden in the native protein. The 42 kDa band could not be detected for the *C214S* and for the *G249S* mutation suggesting that distinct mechanisms might cause the reduced protein levels of these PRPH2 mutants. The moderate decrease of protein expression of the *G249S* mutant is probably caused by a reduction of the relative amount of the correctly spliced PRPH2 transcript due to the generation of a novel donor splice site, as shown in Fig 2A. Accordingly, the resulting *G249S* mutant protein bears a deletion of 30 amino acids covering the most distal part of the D2 loop domain and the proximal half of transmembrane domain 4 (Fig 2B and S2 Fig panel B). This deletion is expected to result in a 3.5 kDa shift leading to a calculated molecular mass of 63 kDa. However, no band for the *G249S* mutant was detected at this molecular weight indicating that the aberrantly spliced isoform is degraded either at the mRNA or at the protein level. In summary, despite the remarkable variability of the single disease mechanisms observed for four of the six adRP associated mutants described above, their most common feature is that they all lead to a reduction of protein expression in outer segments of rods. Of note, differences in protein expression shown in Fig 5G and Fig 6H might also result from the individual differences in the size of the injection areas or from differences in viral transduction efficiencies between the constructs. To exclude these possibilities, we performed two sets of experiments. First, using fundus photography, we show that the size of the injected areas was comparable for the single mutants and the WT PRPH2 in rods and cones always covering approx. 1/3 of the complete retina (S4 Fig). Second, we reasoned that potential differences in transduction efficiencies should also lead to differences in the sum of the single transcript intensities between the PRPH2 minigenes obtained from RT-PCR analysis. However, no substantial variability could be detected when comparing the sum of band intensities for the single PRPH2 constructs in rod or cones (S5 Fig). Hence, we conclude that the differential effects of the single PRPH2 mutants on protein expression reflect intrinsic properties of the mutants and are most likely not caused by technical issues.

### Splicing and protein expression of a rod-dominant PRPH2 mutant in cones and a cone-dominant mutant in rods

So far, analysis of splicing and protein expression of PRPH2 mutations associated with rod or cone defects was exclusively conducted in the photoreceptor type reported to be affected by the given mutation. To exclude the possibility that mutations also influence splicing or protein expression in the photoreceptor type that is primarily not impaired by the mutation, we expressed the rod-dominant *W246R* mutation in cones and the cone-dominant *R220W* mutant in rods, respectively. However, protein localization, splicing, and protein expression levels of both mutants appeared normal and were indistinguishable from the corresponding wild type (S6 Fig).

## Discussion

In this study, using minigenes expressing human WT and eleven PRPH2 point mutants in murine photoreceptors, we uncover a novel role of mRNA splicing on PRPH2 protein expression in rods and in cones. RT-PCR based analysis of PRPH2 transcripts in murine retinas transduced with the PRPH2 minigenes identified three different PRPH2 splice isoforms in rods and cones. These splice isoforms comprise the unspliced transcript, the intron 1 retention, and the correctly spliced PRPH2. Importantly, we confirmed the existence of the unspliced isoform by qRT-PCR from native human and murine retina and FAC-sorted murine rods and cones. Furthermore, analysis of the relative abundance of unspliced and correctly spliced PRPH2 isoforms in sorted rods and cones suggests that, compared to rods, cones express lower amounts of the correctly spliced and higher amounts of the unspliced PRPH2 transcript. In addition, we reveal that only the correctly spliced isoform was translated into protein, whereas the remaining two splice isoforms were probably degraded on mRNA level. This indicates that in cones, alternative mRNA splicing might represent a mechanism that has evolved to keep PRPH2 protein expression rather low. Currently, one only may speculate why cones are anxious for lower amounts of PRPH2 and long-term studies (e.g. in knock-in animals carrying the respective cone-dominant mutants) are required to elucidate the exact function of differential expression levels of PRPH2 in photoreceptors. A comparison of the splicing pattern and protein expression of the PRPH2 mutants in rods and cones revealed a novel genotype-phenotype correlation (Table 3). Three of five mutants associated with cone disorders resulted in a robust increase in the relative amount of the correctly spliced PRPH2 transcript compared to the WT in cones. This increase in mRNA splicing efficiency was accompanied by an increase in protein expression as seen in immunostaining and Western blotting from transduced retinas. None of the cone-dominant PRPH2 mutants affected protein localization or protein expression pattern. In a previous study, it was shown that a very moderate overexpression of PRPH2 in cones (50%) does not affect their morphology and function [30]. However, our results imply that some PRPH2 point mutants can trigger very profound overexpression that may well exert detrimental effects on cones. Two of the cone-dominant PRPH2 mutants (*S212T* and *R220W*) analyzed herein did not show any effects on splicing and protein expression. This suggests that protein overexpression is not an universal mechanism sufficient to explain the penetrance of all cone disease-linked PRPH2 mutants. In line with this, a recent study analyzing another cone disease-linked D2 loop mutation in PRPH2 (*R172W*) revealed detrimental effects of this mutation on cones without affecting the protein expression [31]. Further studies are necessary to determine whether protein overexpression on its own, a functional defect caused by the PRPH2 mutation, or a combination of both mechanisms underlie the observed disease phenotype.

**Table 3:** Summary of the effects caused by single mutants analyzed in this study.

	C214S	P210L	S198R	Q226E	W246R	G249S	V209I	R195L	R220Q	S212T	R220W
**Protein expression**	↓↓↓	↓↓	↓↓↓	n.c.	n.c.	↓	↑↑↑	↑↑↑	↑↑↑	n.c.	n.c.
**Protein localization**	IS	IS	OS	OS	OS	OS	OS	OS	OS	OS	OS
**mRNA splicing**	n.c.	n.c.	n.c.	n.c.	n.c.	novel donor splice site	increased splice efficiency	increased splice efficiency	increased splice efficiency	n.c.	n.c.

↑, increase, ↓, decrease in protein expression in comparison to the corresponding wild type.

IS, inner segment

OS, outer segment.

n.c., no change.

In contrast to PRPH2 mutants in cones, four out of six adRP-linked mutants led to a reduction of protein expression in outer segments of rods via different mechanisms. These mechanisms include aberrant mRNA splicing, protein mislocalization, and protein degradation (Table 3). The only mutant affecting the mRNA splicing in rods was the *G249S* mutation. This mutant results in the generation of a novel donor site in exon 2, thereby reducing the expression relative to the correctly spliced PRPH2 by approximately 20%. Notably, only the correctly spliced PRPH2 was expressed on protein level, whereas the remaining two isoforms appeared to be degraded on mRNA level, presumably via the NMD mechanism. In a previous study in transgenic mice, it was shown that the critical level of PRPH2 expression required to maintain the correct morphology and function of rods is about 80% of the native PRPH2 expression [30]. Thus, the 20% reduction of protein expression induced by the *G249S* mutation is very close to this critical level and might be sufficient to cause retinal degeneration in the affected patient.

Recently, it was shown that mRNA splicing of BBS8, a gene whose defects are associated with Bardet-Biedl syndrome, is different in rods and cones [32]. This finding and the results of our study imply that in rods and cones, components of the splicing machinery are dissimilar. It appears conceivable that cones express a subset of exonic/intronic splice enhancers or silencers which are not present in rods and vice versa. In accordance with this hypothesis, some known ESEs (such as SC35 encoded by the SFRS2 gene), but also the ESS hnRNPA1 were found to be higher expressed on transcript level in rods compared to cones [33] (http://www.fmi.ch/roska.data/index.php). In addition to the transcript data, a careful analysis of the proteome of rods and cones could help identifying molecular components which mediate the differential splicing in rods and cones. Since deletion of intronic regions can also lead to disruption of existing intronic splice regulatory elements [34, 35], we cannot exclude the possibility that the lack of the full-length native introns in PRPH2 minigenes used in this study might also affect the mRNA splicing. The length of the native PRPH2 introns by far exceeds the cloning capacity of viral vectors commonly used for the transduction of photoreceptors. Alternative approaches for insertion of large transgenes to photoreceptors (i.e. electroporation) are very limited in terms of their efficiency [13]. However, high efficiency is crucial for expression analysis in cones, which account for a very small percentage of the photoreceptor population. Thus, from a technical point of view, the rAAV-mediated gene transfer currently appears the most suitable method for analysis of splicing and protein expression in photoreceptors.

To our best knowledge, this is the first study showing an experimental design that allows for the systematic analysis of disease associated point mutations on splicing and on protein expression in photoreceptors for a single gene. Our results suggest that differential splicing of PRPH2 in rods and cones might influence the disease mechanisms of single point mutations on transcript level. Thus, the potential effects of point mutations on splicing in rods and cones might have been underestimated so far and should be tested for other point mutations in PRPH2 but also for mutations in other genes involved in structural and functional integrity of photoreceptors. Finally, the differential impact of PRPH2 point mutations on protein expression in outer segments might help to explain the diverse penetrance of single PRPH2 mutations in rods and cones.

## Materials and Methods

### Ethics statement

Human post mortem retina samples used in this study originate from organ donors with explicit informed consent and the approval by the local ethics committee for the use of tissue for research purposes.

### Animals

All procedures concerning animals were performed with permission of local authorities (Regierung von Oberbayern). Anesthesia was performed by subcutaneous injection of ketamine (40mg/kg body weight) and xylazine (20 mg/kg body weight). Euthanasia was performed by cervical dislocation.

### Construction and cloning of the PRPH2 minigenes

To generate the PRPH2 minigenes, three overlapping products containing the DNA-fragments with full-length exons and shortened PRPH2 introns were PCR amplified from the human genomic DNA isolated from one of the authors of this study (Elvir Becirovic). The PCR fragments were assembled using the standard overlap PCR techniques and subcloned to the pcDNA3.1 expression vector (Invitrogen). Subsequently, citrine was subcloned 5’ to exon 1 of PRPH2. The single PRPH2 point mutants were obtained by site directed mutagenesis (QuikChange Lightning Mutagenesis Kit, Agilent Technologies) according to manufacturer’s instructions. For expression in rods and cones, the single PRPH2 minigenes were subcloned to the pAAV2/8 vector containing the human rhodopsin (hRHO) or the mouse S-opsin (mSWS) promoter. Total length of the insert between the inverted terminal repeats (ITRs) including the promoter, PRPH2 minigene, and the BGH polyA sequence was 4.7 kb for the hRHO promoter containing vector and 4.4 kb for the pAAV vector bearing the mSWS promoter. All PRPH2 minigenes were completely sequenced prior to use.

### In silico prediction of splicing

To predict the splicing of the single PRPH2 point mutants, a free trial ASSEDA splice software (splice.uwo.ca/) and open access NNSplice (Berkeley Drosophila Genome Project, http://www.fruitfly.org/seq_tools/splice.html) software were used.

### Splice analysis and quantification of PRPH2 minigene-derived isoforms in rods and cones

For splice analysis in rods and cones, mice were injected on P14. Three weeks post injection of the respective PRPH2 minigenes, retinas were scanned for fluorescence by means of fundus photography (Spectralis, Heidelberg Eye Instruments). Subsequently, four of the dissected fluorescent retinas for each PRPH2 minigene were pooled and used for RNA isolation. The RNA isolation from injected photoreceptors was performed using the RNeasy Mini Kit (Qiagen) according to the instructions of the manufacturer. Equal concentrations (1 μg) of the isolated total RNA from each sample were applied for the subsequent cDNA synthesis (Revert Aid First Strand cDNA Sythesis Kit, Life Technologies). To amplify the splice isoforms resulting from the PRPH2 minigenes via PCR, following primers were used:

5’-GGCATGGACGAGCTATACAAG-3‘

5’-GGTTGGACACACCATCCAGCG-3‘

The single PCR products representing the different splice isoforms were extracted, purified, and sequenced. All experiments in single cell types concerning the total RNA isolation, cDNA synthesis, and subsequent PCR were performed on the same day under the same conditions. For semi-quantitative analysis of the single PRPH2 minigenes, five RT-PCR reactions were conducted using the cDNA described above with variable number of cycles ranging between 25–27 cycles for amplification of PRPH2 minigenes harboring the hRHO promoter, and 30–32 cycles for amplification of PRPH2 minigenes bearing the mSWS promoter, respectively. Statistical analysis was done with one-way ANOVA followed by the Dunett’s test for multiple comparisons of the single PRPH2 mutants to the corresponding WT PRPH2 in rods and cones. The absolute intensities of the single splice bands were determined using the Image Lab software (BioRad). The experimental conditions and the type of data analysis for the semi-quantitative RT-PCR analysis of the single PRPH2 minigenes shown in S6 Fig were identical to those described for Fig 2 except for the number of technical replicates (three in S6 Fig and five in Fig 2).

### rAAV production and subretinal injections

The PRPH2 minigenes were subcloned to the single stranded pAAV2.1 vector containing either the hRHO or mSWS promoter [36, 37]. Detailed procedures of rAAV production and subretinal injections were described previously [36]. Titer-matched rAAVs (10^9^ particles/μl) containing the single PRPH2 minigenes were subretinally injected to WT C57 BL/6J animals on postnatal day 14. Three weeks post injection, all injected retinas were analyzed for the fluorescence using the scanning laser ophthalmoscopy (Spectralis, Heidelberg Eye Instruments).

### Immunohistology and western blotting

For immunohistology, mice were injected at P14 with the respective PRPH2 minigenes. Three weeks post injection, the retinas were dissected and processed for immunohistology as described [38]. We used the rabbit anti-CNGB1a (1:2000, [39]) and rabbit anti-M-opsin antibody (1:300, [38]) as marker for rod and cone outer segments, respectively. Retinal images were obtained by the TCS SP8 confocal scan microscope (Leica), acquired with the LASAF software (Leica), and processed with the ImageJ software (National Institutes of Health). For protein detection via Western blotting for each of the constructs, we used four pooled retinas from four mice injected on P14 with the titer-matched PRPH2 minigenes harboring the hRHO or mSWS promoter. Membrane preparations were performed three weeks post injection as described [40] and proteins were separated on a 6–12% SDS PAGE gradient gel. To detect the citrine-tagged proteins, the monoclonal mouse anti-GFP antibody was used (1:1000, Clontech). For detection of the Na^+^/K^+^ ATPase, we used the mouse anti-a6F-c antibody in a 1:1000 dilution (Developmental Studies Hybridoma Banks, University of Iowa). For semi-quantitative analysis, three technical replicates were conducted from membrane preparations. The absolute intensities of the single protein bands were determined using the Image Lab software (BioRad). The band intensities for each minigene-derived PRPH2 protein were normalized to the corresponding ATPase intensity.

### FAC-sorting of rods and cones

Retinas were isolated from neural retina leucine zipper- (Nrl) EGFP [26] or cone-GFP [27] reporter mice. In Nrl-EGFP mice, EGFP is driven by the rod-specific Nrl promoter. In cone-GFP mice, GFP expression is driven by a human red/green opsin gene 5' regulatory sequence. All animal experiments were conducted in strict accordance with EU and German laws (Tierschutzgesetz) and the ARVO Statement for the Use of Animals in Ophthalmic and Vision Research. From each reporter mouse, two retinas were isolated at adult stage (6–8 weeks) and dissociated using Papain Dissociation System (Worthington Biochemical Corporation, Lakewood, USA) for 20 minutes with 100 μg/ml papain following manufacturer’s instructions. Dissociated cells were sorted (BD FACSAria II SORP, BD Bioscience) and cells were collected based on their reporter fluorescence.

### RNA isolation and cDNA synthesis

RNA from FACS purified rod and cone photoreceptors and from native human and murine retina was extracted using RNeasy Minikit (Qiagen, Hilden, Germany) according to the manufacturer’s instructions. RNA concentration and purity was determined using NanoDrop2000 (Thermo Scientific). For cDNA synthesis from native human (two donors) and murine retina (four animals), an amount of 500 ng total RNA was used. For cDNA synthesis from sorted murine rods and cones, 50 ng of isolated total RNA was applied.

### Quantitative PCR

PCR was performed on a StepOnePlus Real-Time PCR System (Applied Biosystems) using SYBR Select Master Mix (Applied Biosystems). For quantitative PCR, we performed three technical replicates for each gene and normalized each of them to the expression of the housekeeping gene aminolevulinic acid synthase (Alas). For detection of the murine transcripts, following primers were used:

Alas fwd: 5’-TCGCCGATGCCCATTCTTATC-3‘

Alas rev: 5’-GGCCCCAACTTCCATCATCT-3‘

Rhodopsin fwd: 5’-GCCTCGAGAGCCGCAGCCATG-3‘

Rhodopsin rev: 5’-GCAGGAACATGTACGCTGCC-3‘

M-opsin fwd: 5’-GTTCCAGAGACAGTTTTCTAC-3‘

M-opsin rev: 5’-CAACGACCACAAGAATCATCC-3‘

mP-cs_F: 5’- TCTCCTCCAAGGAGGTCAAAG -3‘

mP-cs_R: 5’- GAGTCCGGCAGTGATGCTCAC -3‘

mP-us_F: 5’- GGGAGGATCTGCTGCTTGGTG -3‘

mP-us_R: 5’- GCTCACCAGGTCTGTCTTCAC -3‘

For detection of the human transcripts, following primers were used:

ALAS fwd: 5’-GATGTCAGCCACCTCAGAGAAC-3‘

ALAS rev: 5’-CATCCACGAAGGTGATTGCTCC-3‘

hP-cs_F: 5’-GTGGATCAGCAATCGCTACC -3‘

hP-cs_R: 5’-GGTTGGACACACCATCCAGCG -3‘

hP-us_F: 5’-GAAGTGGCCCCTGTTGAGAAG -3‘

hP-us_R: 5’-CATTAGACCCAAATGGGACCG -3‘.

## Supporting Information

S1 Fig Exon 2 specific disease associated point mutations in PRPH2. Left, the region of PRPH2 encoded by exon 2 is shown as a dashed rectangle. Right, schematic magnification of the exon 2-encoded part. Currently known positions of point mutations are highlighted in green. Arrowheads point to the mutations affecting the amino acids analyzed in this study. Among those, black arrowheads highlight the mutations linked to cone diseases, whereas the red arrowheads point to the adRP-associated mutations.
(TIF)

S2 Fig Representative sequence analyses of PRPH2 splice products. (A) Sequencing results of the exon boundaries from the correctly spliced PRPH2 transcript. Bottom left, topology of the correctly spliced PRPH2. The region encoded by exon 2 is given as a dashed rectangle. N, N-terminus, C, C-terminus. (B) Sequencing results of the *G249S* mutant. Upper panel, scheme showing the *G249S* mutant before (top) and after splicing (bottom). Lower panel, left, predicted impact of the aberrantly spliced *G249S* on protein level. The generation of a novel splice donor site (DS) leads to an in-frame deletion of 30 amino acids (aa) covering the part of PRPH2 symbolized by the brown transparent rectangle. Middle and right, electropherograms showing the impact of the *G249S* mutant before and after splicing. The position of the novel splice donor site is shown as an arrow and the position of the mutation is symbolized by an arrowhead. (C) Retention of intron 1 and the unspliced PRPH2 transcript result in a frameshift followed by a stop codon immediately after exon 1. The corresponding protein lacks the distal half of the D2 loop, the transmembrane domain 4, and the C-terminus (bottom left). All sequencing reactions were performed on bands isolated from murine retina after viral transfection with PRPH2 minigenes (Fig 2).
(TIF)

S3 Fig Determination of the linear amplification range of RT-PCR for PRPH2 minigenes. Representative qRT-PCR from retinas injected with WT PRPH2 minigenes harboring a rod hRHO (rP-mg) or cone mSWS (cP-mg) specific promoter. For qRT-PCR, the same pooled retina samples and the same primers were used as described in Fig 2B. The dashed lines represent the window of cycles falling within the linear amplification range for rP-mg (20–28 cycles, magenta) and cP-mg (26–33 cycles, green), respectively.
(TIF)

S4 Fig Representative fundus photography of murine retinas injected with single PRPH2 minigenes. Fundus photography images were performed three weeks post injection on wild type mice injected at the age of two weeks with the single PRPH2 minigenes under the control of a cone specific mSWS (A) or rod specific hRHO (B) promoter. Scale bar represents 800 μm.
(TIF)

S5 Fig Sum of absolute band intensities for PRPH2 minigenes in rods and cones. The data shows the total sum of absolute intensities of all bands detected for each PRPH2 minigene from five technical replicates shown in RT-PCR experiments in Fig 2A. The bars represent the mean values ± SEM conducted to calculate the relative percentages displayed in Fig 2C–2E. Statistical analysis (PRPH2 mutants *vs* the corresponding WT in rods and cones, respectively) was performed using one-way ANOVA followed by the Dunett’s test. *, p< 0.05; **, p< 0.01; ***, p< 0.001. n.s., not significant.
(TIF)

S6 Fig Cross-expression of one rod-dominant PRPH2 mutant in cones and one cone-dominant mutant in rods. Protein localization (A, F), mRNA splicing (B, C, G, H), and protein expression (D, E, I, J) of a cone dominant *R220W* mutation in rods (A-E) and a rod-dominant *W246R* mutation in cones (F-J). The experimental conditions (age of injected mice, immunolabeling of retinal sections, time point of RNA isolation, number of cycles used for the RT-PCR, number of injected animals, time point of protein isolation, type of data presentation) are identical to those described for the corresponding experiments in Figs 1, 2, 5 and 6. For semi-quantitative analysis of mRNA splicing (C, H) and protein expression (E, J) in rods and cones, three technical replicates were conducted. Statistics were calculated using the two-tailed t-test. p-values shown in C, H, E, and J are as follows: C, p_unspliced_ = 0.91, p_correctly spliced_ = 0.76; H, p_unspliced_ = 0.74, p_correctly spliced_ = 0.87; E, p = 0.39; J, p = 0.25. Scale bar in A, F represents 20 μm.
(TIF)

S1 Table *In silico* splice analysis of 30 point mutations in exon 2 of PRPH2. For splice analysis using ASSEDA prediction software, 40 bp flanking the respective mutation were used. All changes for single splice elements or donor and acceptor sites are shown as fold changes to the WT. For NNSplice based prediction, single scores are given for the WT PRPH2 and changes to this score for the mutants are highlighted in blue. ↑ or ↓, increase or decrease of the score for a given recognition sequence or for a donor and acceptor site, respectively. The numbers next to these symbols represent the fold change to the WT. AS, splice acceptor site, DS, splice donor site, n.c., no change. The position of the respective splice acceptor or donor site (AG and GT, respectively) within the given DNA-sequence is shown in red. SF2/ASF, SC35, SRp40 und SRp55, serine/arginine (SR)-rich proteins belonging to the exonic splice enhancer (ESE). hnRNPA1, proteins belonging to the exonic splice silencer (ESS). Both, abolition of the consensus sequence of ESEs or generation of a novel recognition sequence for ESS, might lead to exon skipping or intron retention.
(XLSX)
