# Supplementary figures and images for: In Vivo Analysis of Disease-Associated Point Mutations Unveils Profound Differences in mRNA Splicing of Peripherin-2 in Rod and Cone Photoreceptors
Source: PLoS Genet. 2016 Jan 21;12(1):e1005811. doi: 10.1371/journal.pgen.1005811 (PMC4722987; doi:10.1371/journal.pgen.1005811)

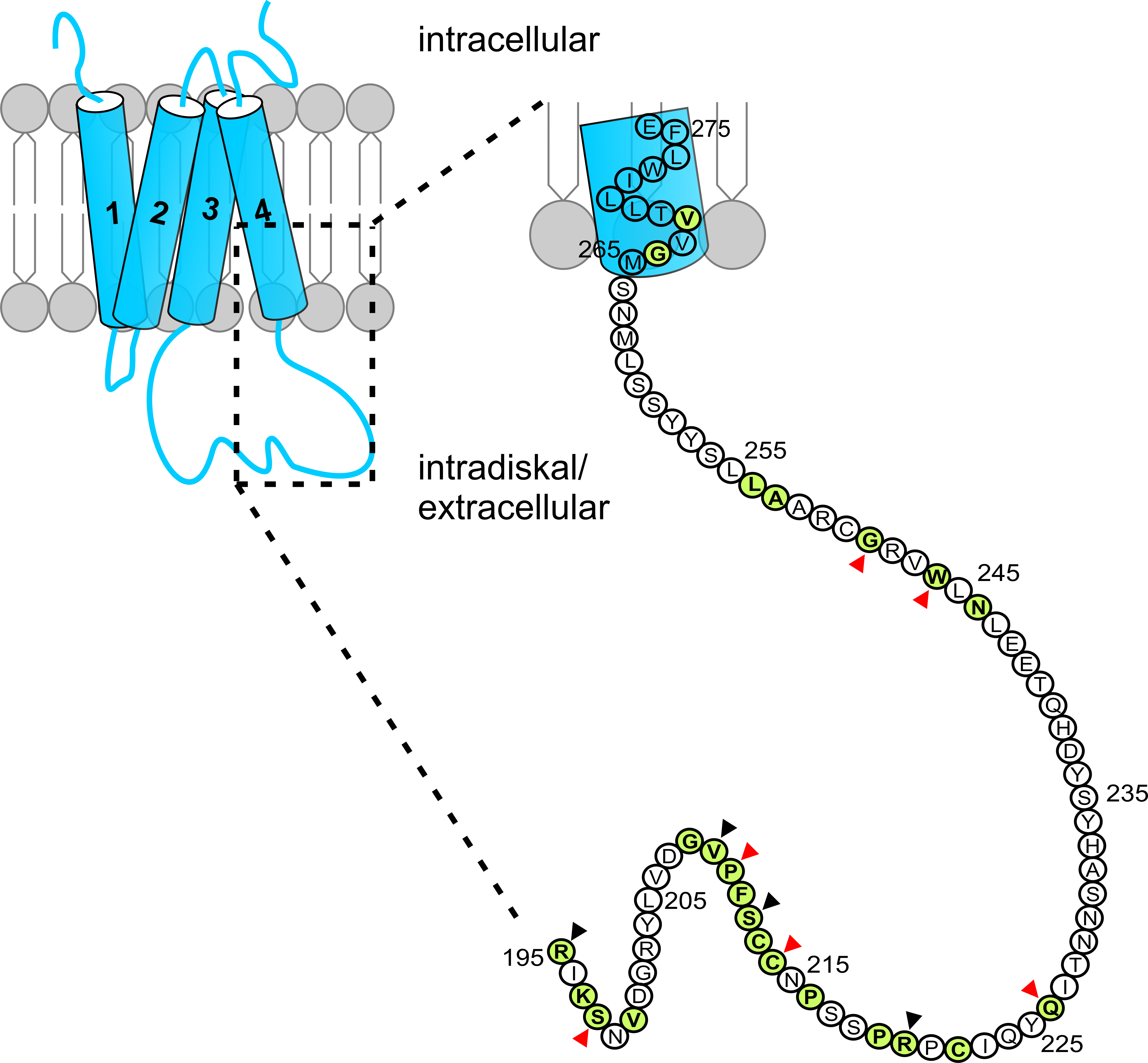

Supplement: S1 Fig — Left, the region of PRPH2 encoded by exon 2 is shown as a dashed rectangle. Right, schematic magnification of the exon 2-encoded part. Currently known positions of point mutations are highlighted in green. Arrowheads point to the mutations affecting the amino acids analyzed in this study. Among those, black arrowheads highlight the mutations linked to cone diseases, whereas the red arrowheads point to the adRP-associated mutations. (TIF) [file pgen.1005811.s001.tif]

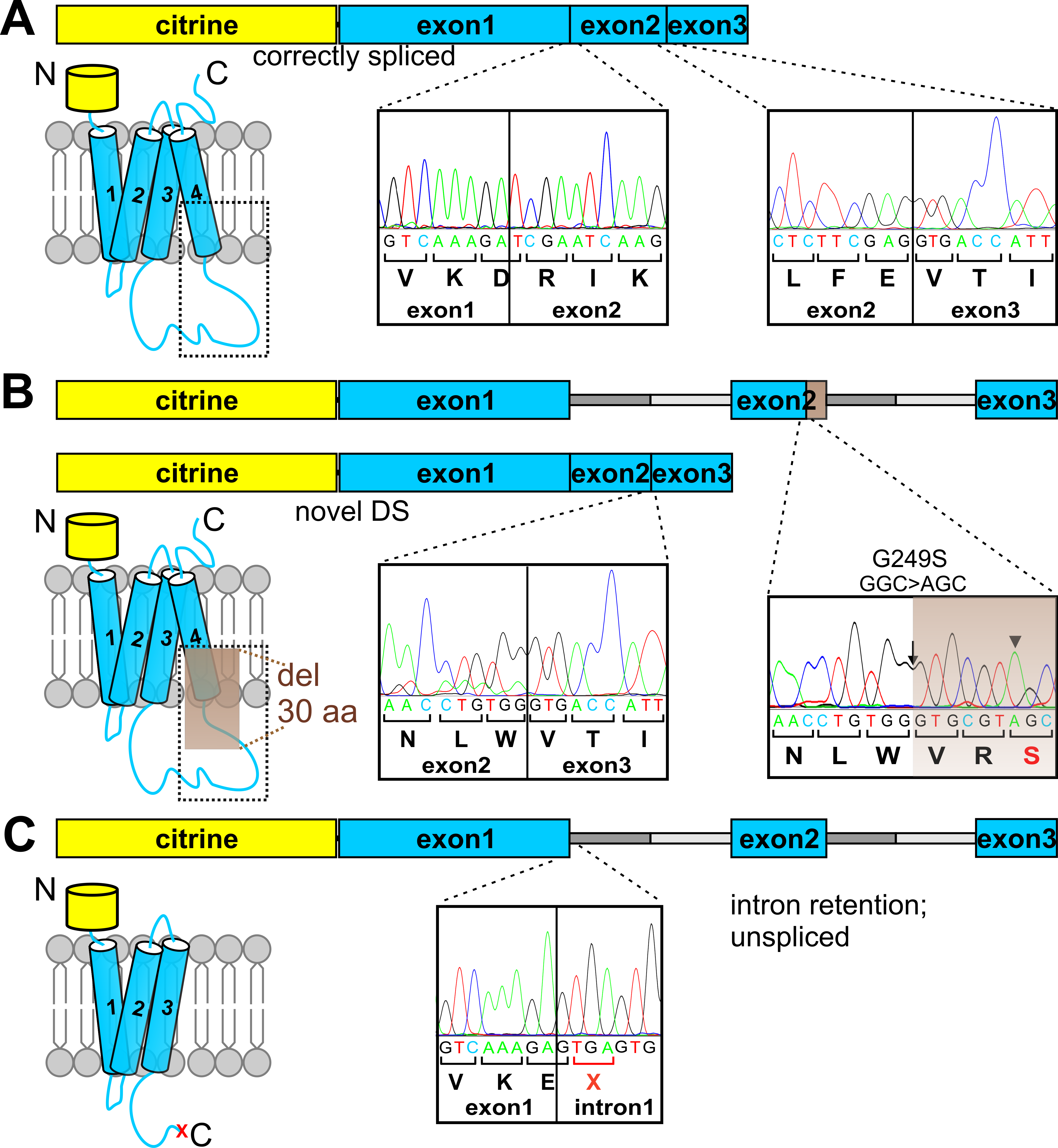

Supplement: S2 Fig — (A) Sequencing results of the exon boundaries from the correctly spliced PRPH2 transcript. Bottom left, topology of the correctly spliced PRPH2. The region encoded by exon 2 is given as a dashed rectangle. N, N-terminus, C, C-terminus. (B) Sequencing results of the G249S mutant. Upper panel, scheme showing the G249S mutant before (top) and after splicing (bottom). Lower panel, left, predicted impact of the aberrantly spliced G249S on protein level. The generation of a novel splice donor site (DS) leads to an in-frame deletion of 30 amino acids (aa) covering the part of PRPH2 symbolized by the brown transparent rectangle. Middle and right, electropherograms showing the impact of the G249S mutant before and after splicing. The position of the novel splice donor site is shown as an arrow and the position of the mutation is symbolized by an arrowhead. (C) Retention of intron 1 and the unspliced PRPH2 transcript result in a frameshift followed by a stop codon immediately after exon 1. The corresponding protein lacks the distal half of the D2 loop, the transmembrane domain 4, and the C-terminus (bottom left). All sequencing reactions were performed on bands isolated from murine retina after viral transfection with PRPH2 minigenes (Fig 2). (TIF) [file pgen.1005811.s002.tif]

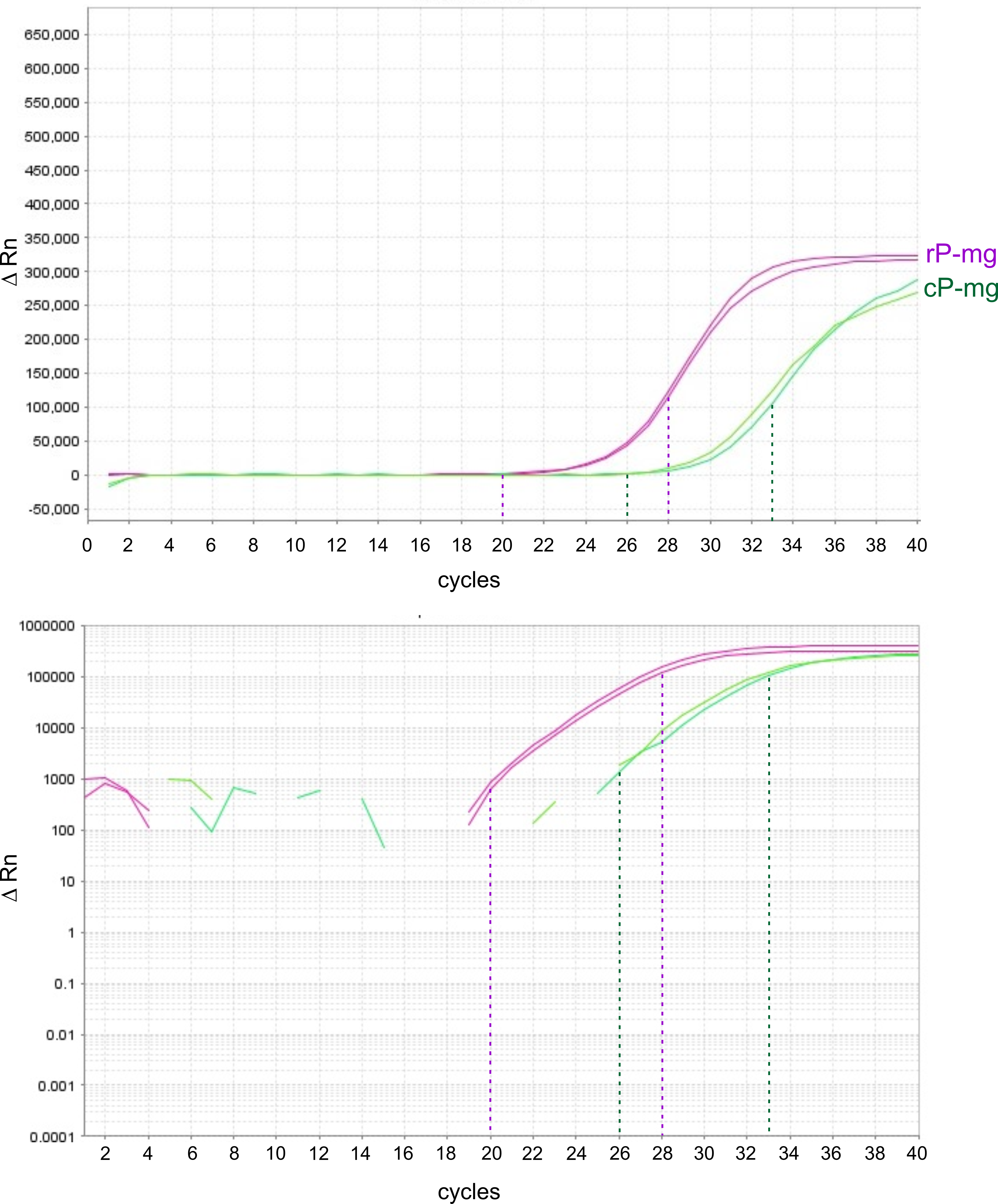

Supplement: S3 Fig — Representative qRT-PCR from retinas injected with WT PRPH2 minigenes harboring a rod hRHO (rP-mg) or cone mSWS (cP-mg) specific promoter. For qRT-PCR, the same pooled retina samples and the same primers were used as described in Fig 2B. The dashed lines represent the window of cycles falling within the linear amplification range for rP-mg (20–28 cycles, magenta) and cP-mg (26–33 cycles, green), respectively. (TIF) [file pgen.1005811.s003.tif]

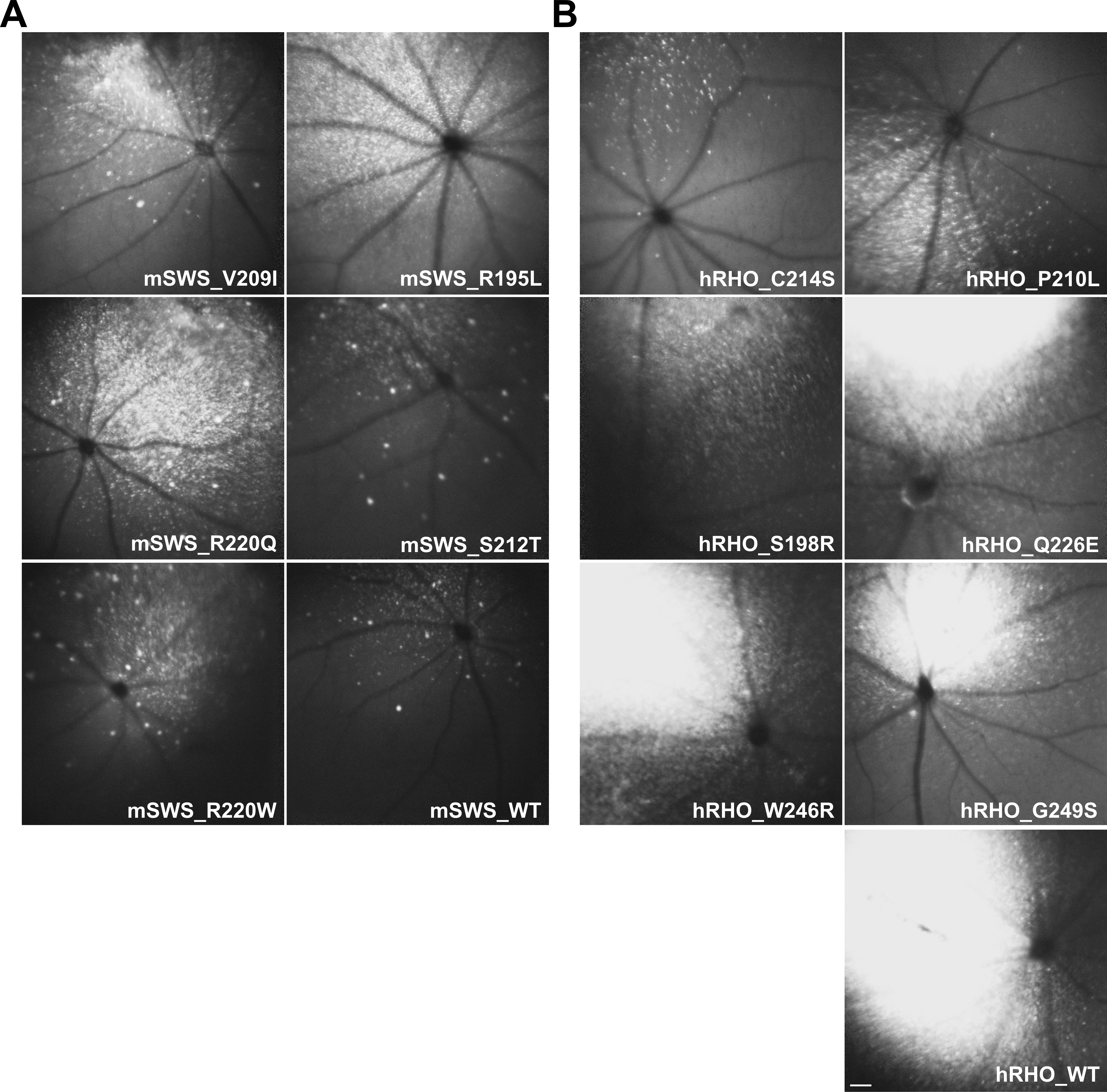

Supplement: S4 Fig — Fundus photography images were performed three weeks post injection on wild type mice injected at the age of two weeks with the single PRPH2 minigenes under the control of a cone specific mSWS (A) or rod specific hRHO (B) promoter. Scale bar represents 800 μm. (TIF) [file pgen.1005811.s004.tif]

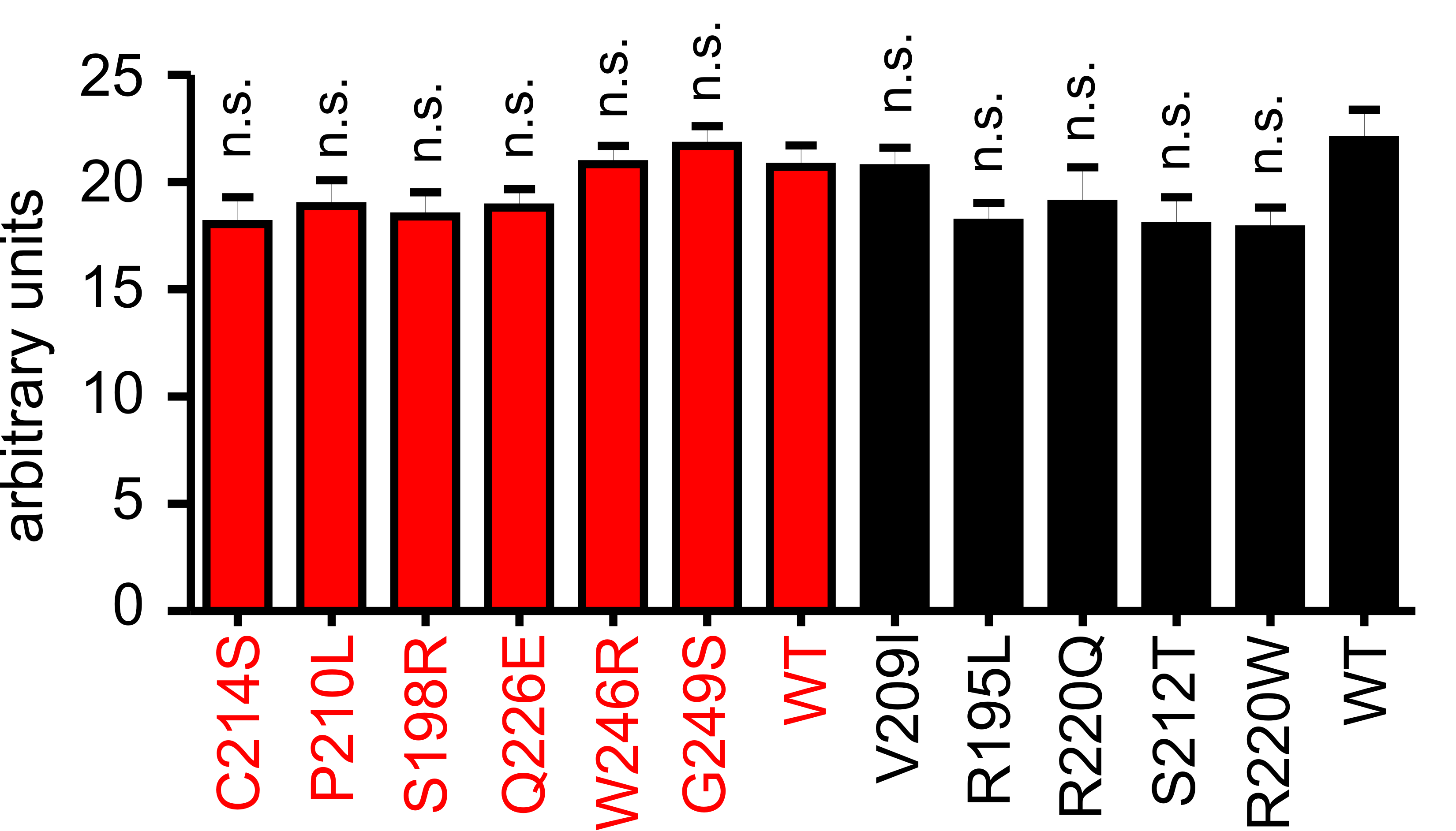

Supplement: S5 Fig — The data shows the total sum of absolute intensities of all bands detected for each PRPH2 minigene from five technical replicates shown in RT-PCR experiments in Fig 2A. The bars represent the mean values ± SEM conducted to calculate the relative percentages displayed in Fig 2C–2E. Statistical analysis (PRPH2 mutants vs the corresponding WT in rods and cones, respectively) was performed using one-way ANOVA followed by the Dunett’s test. *, p< 0.05; **, p< 0.01; ***, p< 0.001. n.s., not significant. (TIF) [file pgen.1005811.s005.tif]

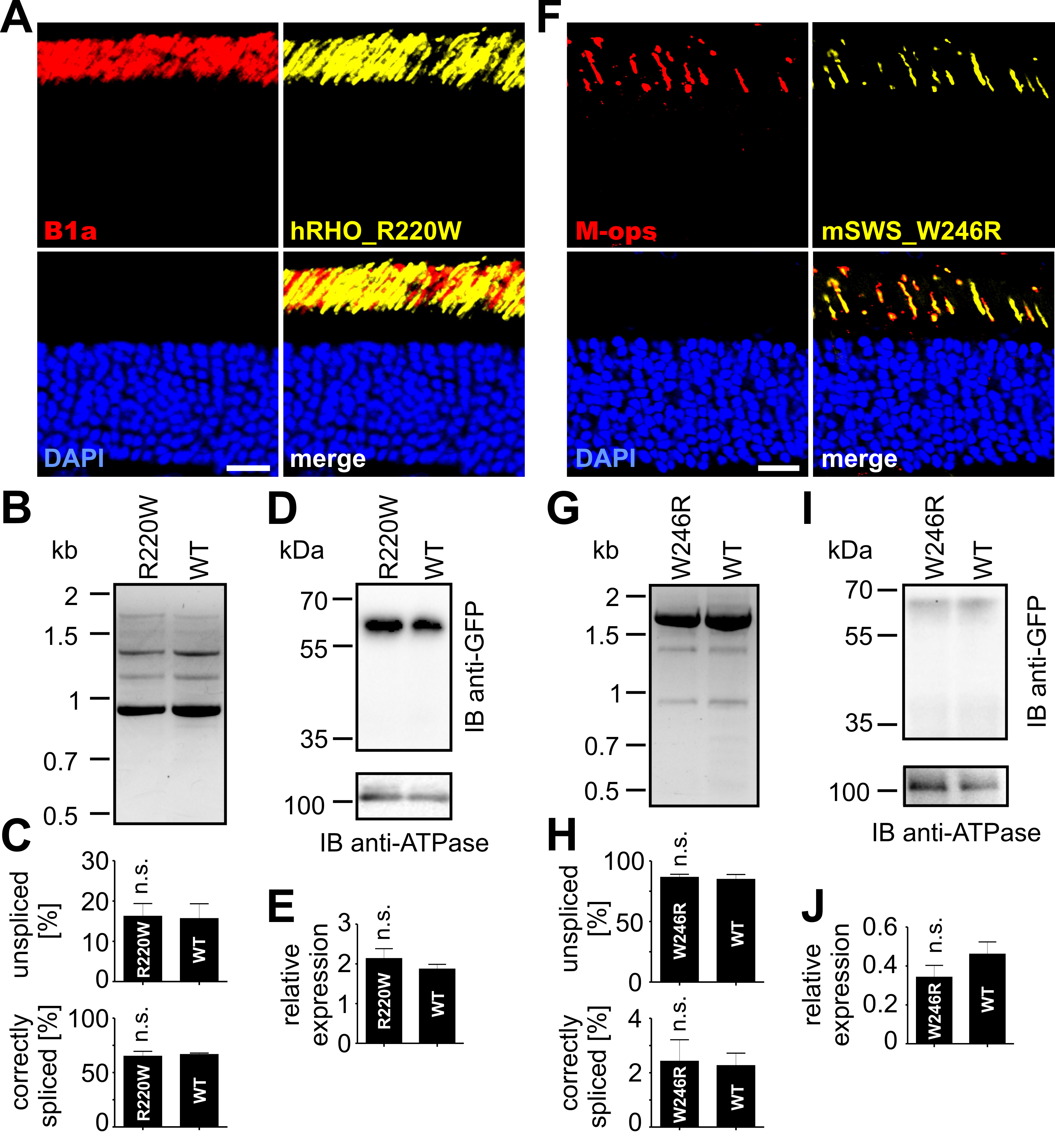

Supplement: S6 Fig — Protein localization (A, F), mRNA splicing (B, C, G, H), and protein expression (D, E, I, J) of a cone dominant R220W mutation in rods (A-E) and a rod-dominant W246R mutation in cones (F-J). The experimental conditions (age of injected mice, immunolabeling of retinal sections, time point of RNA isolation, number of cycles used for the RT-PCR, number of injected animals, time point of protein isolation, type of data presentation) are identical to those described for the corresponding experiments in Figs 1, 2, 5 and 6. For semi-quantitative analysis of mRNA splicing (C, H) and protein expression (E, J) in rods and cones, three technical replicates were conducted. Statistics were calculated using the two-tailed t-test. p-values shown in C, H, E, and J are as follows: C, punspliced = 0.91, pcorrectly spliced = 0.76; H, punspliced = 0.74, pcorrectly spliced = 0.87; E, p = 0.39; J, p = 0.25. Scale bar in A, F represents 20 μm. (TIF) [file pgen.1005811.s006.tif]
